# FERTILITY CARE IN LOW- AND MIDDLE-INCOME COUNTRIES: Policy, politics, and macro-level influences on implementation in Uganda

**DOI:** 10.1530/RAF-24-0063

**Published:** 2025-06-26

**Authors:** Margaret Joanita Mutumba-Nakalembe, Craig R Janes

**Affiliations:** ^1^ Western University, School of Biomedical Engineering, London, Ontario, Canada; ^2^ University of Waterloo, School of Public Health Sciences, Waterloo, Ontario, Canada

**Keywords:** fertility, low-cost IVF, human fertility, in vitro fertilization, external context, Sub-Saharan Africa, implementation process, assisted reproductive technologies, macro-context, consolidated framework for implementation science

## Abstract

**Graphical Abstract:**

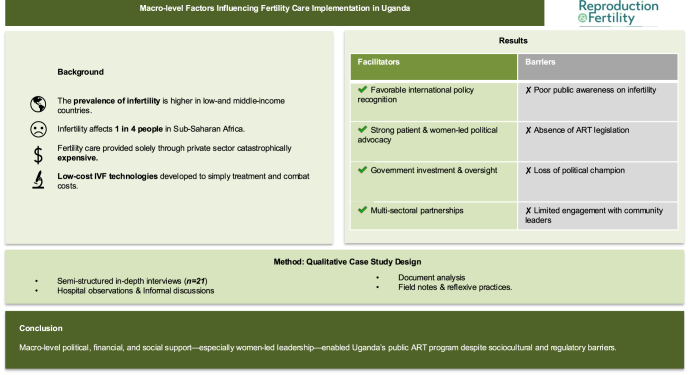

**Abstract:**

**Background**

Infertility is a reproductive disease affecting millions globally. In Sub-Saharan Africa, the burden is considerably higher, affecting one in four couples. The psychosocial and economic impacts of infertility remain severe. Furthermore, restricted access to affordable fertility services is justified by international population reduction agendas and limited resources, resulting in inequitable access. Treatment, when available, is primarily through private sector clinics at catastrophically high costs. For this reason, low-cost IVF (LCIVF) technologies have been developed to simplify and minimize treatment costs. Still, there are limited studies on their adoption and utilization in the region.

**Methods**

A qualitative case study was used to explore implementation of LCIVF technologies in Uganda’s public health system. Macro-level factors influencing implementation of an ART department at Mulago Women’s Hospital were assessed through semi-structured interviews conducted with 21 actors, along with hospital observations, field notes, and document review. A combination of inductive and deductive thematic analysis techniques was used for data analysis in NVivo 12, guided by the Consolidated Framework for Implementation Research (CFIR).

**Results**

Following our analysis, several factors facilitated macro-level implementation including acknowledgment of infertility as a reproductive disease, strong political advocacy and oversight, government funding, and multi-organizational collaboration. Barriers included poor public knowledge, absence of legislation, limited community leader engagement, and diminished political support.

**Contributions**

This study contributed to knowledge on external factors that influence sustainable implementation of LCIVF initiatives in low-resource settings and is one of the first studies to apply CFIR to fertility care implementation in a low-resource setting.

**Lay summary:**

Infertility is a reproductive disease that makes it difficult to conceive a child. Globally, infertility impacts millions of people. In Sub-Saharan Africa, the rate of infertility is surprisingly higher. There are few centers providing affordable treatment. This is partly because population reduction is an international priority, neglecting many who cannot have even one child. Cheaper treatment options have been developed to make treatment more accessible and affordable. This study looked at how international and national politics influenced inclusion and access to fertility care in an African context. A qualitative case study design approach was used. Our analysis found international acceptance of infertility as a disease, strong political support, and financial support aided inclusion. Still, limited public knowledge, engagement of community leaders, and diminished political support slowed down efforts. This research suggests that international agendas, political support, and community engagement are needed for sustained inclusion of fertility services in low-income countries.

## Introduction

Context matters when introducing new, complex interventions in any health system (Nilsen & Bernhardsson 2019). In this study, we examined how macro-level contextual factors influenced implementation of fertility services into the public health system of a Sub-Saharan African context. Infertility is a reproductive disease of global public health concern, affecting an estimated 186 million people worldwide (The Lancet Global Health 2022). Notably, low- and middle-income countries (LMICs) exhibit the highest prevalence of infertility. In Sub-Saharan Africa (SSA), infertility prevalence is as high as 30–40% (WHO 2010, Mascarenhas *et al.* 2012, Inhorn & Patrizio 2015, Legese *et al.* 2023), translating to one in every four couples of reproductive age compared to the global average of one in six (Vander Borght & Wyns 2018, Chiware *et al.* 2021, WHO 2023). The main causes of infertility in SSA include untreated sexually transmitted infections, reproductive tract infections, poor obstetric and postpartum care, unsafe abortions, and harmful cultural practices (Ombelet & Onofre 2019). In this region, the psychosocial consequences of childlessness are often more severe than in developed countries, as families depend on children for economic survival and continuation of their lineage, among other factors. The inability to have children, particularly for women, leads to isolation, ostracization, increased sexually transmitted disease (STD) risk, intimate partner violence, divorce, polygamy, economic instability, and suicide in some cases (Cousineau & Domar 2007, WHO 2013, Anokye *et al.* 2017, Ombelet & Onofre 2019, Roomaney *et al.* 2024). Infertility, therefore, is more than an individual disease in Sub-Saharan Africa. It is both a social, economic, and public health issue.

### Infertility and macro-level drivers

Infertility in Sub-Saharan Africa has long been neglected as a reproductive disease. Historically, infertility was overlooked due to an international focus on reduction of the high fertility rates in resource-limited countries. Global concern for overpopulation and population control led to de-prioritization of infertility in Sub-Saharan Africa, with an emphasis on family planning and contraception interventions (Ombelet 2011, Chiware *et al.* 2021). However, concern for population reduction and economic growth ought not to discount the burden of infertility and the need for affordable treatment for infertile persons (Gribble & Bremner 2012). Denying barriers to family forming and, therefore, access to fertility care is not a legitimate policy solution for population control (Ombelet 2011).

De-prioritization of infertility and access to care in global discourse has been further justified by the high cost of assisted reproductive technologies (ART) needed to overcome infertility, relative to limited resources in low- and middle-income countries. Current treatment modalities, primarily provided in private fertility clinics in SSA are expensive and exclusive to the financially advantaged. The average cost of *in vitro* fertilization (IVF)-related treatment is variable. For instance, in Nigeria, it can be as high as USD $10,000, compared USD $4,000–5,000 in Mali and USD $3,500 in Uganda (Platteau *et al.* 2008, Okohue *et al.* 2010, Fadare & Adeniyi 2015, Hörbst 2016). In Ghana, the cost of one IVF cycle was found to be the equivalent of a nurse’s salary over 18 months (Gerrits & Shaw 2010). This presents significant financial barriers for many, with the cost burden often falling predominantly on women seeking fertility care. Therefore, low- and middle-income countries cannot justify expensive fertility treatments over urgent health needs.

Limited to no ART legislation and regulation of the fertility care industry in many African countries is an additional challenge to service provision (Chiware *et al.* 2021). The absence of ART legislation in Sub-Saharan Africa can partly be explained by the exportation of ART to Africa in the 1980s without the establishment of data registries to monitor and report utilization and outcomes (Botha *et al.* 2018). Yet, ART regulation could enable transparency and treatment safety through licensure, monitoring, and accreditation of fertility providers (IFFS 2016). In addition, formulation of specific fertility care legislation is vital for recognition and incorporation of fertility care into national reproductive health policies and standards of practice (Ombelet 2009, WHO 2010. However, variability in regulation globally has left governments in need of determining their own applications of ART based on cultural norms and local stakeholders.

Finally, poor knowledge of infertility and its treatment modalities by the general population inhibits demystification of the disease and consequential support from key stakeholders such as policymakers and community leaders. This is important because interpretations of infertility in SSA are variable, taking on cultural, naturalistic, and religious concepts. Assumptions of spiritual possession, witchcraft, or punishment have been found in several studies in Africa (Tabong & Adongo 2013, Aluko-Arowolo & Ayodele 2014). These influences, along with a lack of knowledge about ART, are a barrier to early diagnostic acceptance and implementation of ART in Africa (Gerrits 2016, Serour *et al.* 2019, Afferri *et al.* 2022, Bittaye *et al.* 2023).

Therefore, the myriad macro-level factors including limited attention and knowledge on infertility, prioritization of population control, constrained resources, and absence of ART regulation have led to significant inequities in access to fertility care in Sub-Saharan Africa.

### Making fertility care affordable: low-cost IVF initiatives

In recent years, international bodies such as the World Health Organization (WHO) have recognized the impact of infertility and have called for fertility care for all through a holistic, integrated, and comprehensive approach, from the community level through to the health system (WHO 2010). Furthermore, researchers have developed low-cost IVF (LCIVF) technologies to combat the high cost of treatment, one of the most significant barriers to incorporation of fertility care within reproductive health services in Sub-Saharan Africa. By simplifying conventional IVF technologies, LCIVF initiatives intend to reduce the cost of treatment for infertile individuals and improve accessibility in low-and middle-income countries (Ombelet *et al.* 2008, Ombelet 2011, Inhorn & Patrizio 2015). Some LCIVF initiatives consist of simplified diagnostic protocols, minimum ovarian stimulation, simplified laboratory procedures, cheaper incubators, and single embryo transfer (Ombelet 2011, Lucena *et al.* 2012, Nagulapally *et al.* 2012, Bahamondes & Makuch 2014, Lee *et al.* 2017). Although low-cost technologies such as the simplified IVF system have been proven to be safe and effective, there has been limited integration of these technologies in clinical practice (Ombelet *et al.* 2025, 2023). While studies have been conducted on the effectiveness of these technologies, those examining the implementation, access, and affordability of LCIVF services in low-income countries are non-existent (Chiware *et al.* 2021).

This paper responds to the need for in-depth analysis of opportunities and limitations of implementation and impact of low-cost ART in African settings on access to affordable fertility care (Chiware *et al.* 2021, Afferri *et al.* 2022). It is part of a larger case study that explored the multi-level (macro-, meso-, and micro-) factors influencing implementation of affordable fertility care within the public health system of a Sub-Saharan African context – Uganda. Uganda is one of the first countries in the East African region to incorporate fertility care within its public health system for the provision of safe, affordable, and accessible fertility care to its population, was the study site of choice (Oketch 2017). In this paper, the specific objective considered is as follows: ‘How have macro-level factors influenced the implementation of affordable fertility care in Uganda’s public health system?’. We report on the macro-level, broader barriers and facilitators to inclusion of fertility care in Uganda’s public health system using the Consolidated Framework for Implementation Science (CFIR) (Damschroder *et al.* 2009). To our knowledge, this is one of the first analyses in the study of implementation of affordable fertility services in a low-resource country and adds to the application of CFIR.

## Materials and methods

This study was granted ethics approval from The University of Waterloo’s Office of Research Ethics (ORE) (#ORE 42165), Mildmay Uganda Research Ethics Committee (MUREC) (# REC REF 1009–2020), and the Uganda National Council for Science and Technology (UNCST) (#HS1214ES).

The study purpose was to examine macro-level factors that influenced implementation of fertility care within Uganda’s public health system. This was a qualitative, single case study that sought to examine the macro-level barriers and facilitators to installation of Mulago Specialized Women and Neonatal Hospital’s Assisted Reproductive Technology Department.

### Contextual background

Uganda is a low-middle-income country located on the Equator and situated in East Africa. Its population is 45.9 million, relatively young, with less than 4% aged over 65 years and over half (54%) of the population under 18 years of age (UBOS 2024). Most people live in rural areas (73%) in comparison to urban areas (26.6%), and the median monthly household income is USD $53 (UNHS 2020). There has been a notable reduction in Uganda’s fertility rate to 4.7 children per woman in 2022 compared to 7.1 children per woman in 1980 (World Bank 2022).

Paradoxically, Uganda is also recognized among countries with the highest infertility rates, with as many as 30% of couples struggling with this reproductive disease (WHO 2010). The most common type of infertility in the region is secondary infertility due to tubal infections (Kudesia *et al.* 2018). Fertility services are offered mainly through the private sector, with limited regulation and an average cost of USD $4,000 per cycle, paid out of pocket (Sajjabi 2008).

This study took place at Mulago Specialized Women & Neonatal Hospital (hereafter known as Mulago Women’s Hospital – MWH), the first publicly funded, highly specialized, women’s national referral hospital to offer ART services within the reproductive medical care services. The price of consultation and ART counseling is the equivalent of $13 per person, while IVF treatment costs from $3,500–$6,000, with the hospital citing its prices as 60% cheaper than other hospitals in the country (Daily Monitor 2018, New Vision 2018, Waswa 2018). See Table 1 for full list of service prices. Other specialized services include gynecological oncology, high-risk antenatal & postnatal services, and urogynecology (mainly obstetric fistula). MWH aims to service various Sub-Saharan countries including Cote d’Ivoire, Ethiopia, Gambia, Ghana, Kenya, Liberia, Rwanda, Sierra Leone, Tanzania, and Zambia (Oketch 2017).

**Table 1:** Service price list at Mulago Women’s Hospital in Uganda shillings and US dollars (Daily Monitor 2018, New Vision 2018, Waswa 2018).

No.	Service	Price (Uganda shillings)	Price (USD)
1	Consultation fee per visit	UGX 50,000	$13.51
2	Antenatal package (up to eight visits)	UGX 890,000	$240.54
3	Accommodation per day	UGX 80,000	$21.62
4	Nurses/doctors care per day	UGX 100,000	$27.03
5	Normal delivery (SVD)	UGX 800,000	$216.22
6	Caesarean section	UGX 2,000,000	$540.54
7	Additional tubal ligation	UGX 200,000	$54.05
8	Hysteroscopy	UGX 1,500,000	$405.41
9	Laparoscopy	UGX 1,500,000	$405.41
10	Labor with analgesia	UGX 1,600,000	$432.43
11	Cervical cerclage	UGX 250,000	$67.57
12	Neonatal follow-up/immunization	UGX 70,000	$18.92
13	ART counseling	UGX 50,000	$13.51
14	*In-vitro* fertilization	UGX 13,000,000–22,000,000	$3,571 – $6,000

### Theoretical framework

For the larger study, several Implementation Science frameworks were considered. The Consolidated Framework for Implementation Research (CFIR) was employed to understand determinants salient to the inclusion of low-cost IVF in Uganda’s public health system. This framework was selected owing to its comprehensive constructs useful within exploratory research for classification of facilitators and barriers to implementation. The CFIR framework consists of five domains, namely, i) Intervention characteristics, ii) Outer setting, iii) Inner setting, iv) Individual characteristics, and v) Process by which the intervention is achieved (Damschroder *et al.* 2009). These domains have been summarized in Table 2. For the current article, the CFIR was used to facilitate exploratory analysis into the macro-level factors influencing implementation of affordable, low-cost IVF initiatives within the public health system of Uganda, guiding data collection, thematic coding, and analysis.

**Table 2:** Explanation of Consolidated Framework for Implementation Research (CFIR) constructs (Damschroder *et al.* 2009).

Domain	Construct
Intervention characteristics	Intervention source
	Evidence strength and quality
	Relative advantage
	Adaptability
	Trialability
	Complexity
	Design quality and packaging cost
Outer setting	Patient needs and resources
	Cosmopolitanism
	Peer pressure
	External policy and incentives
Inner setting	Structural characteristics
	Networks and communications
	Culture
	Implementation climate
	i) Tension for change ii) compatibility iii) relative priority iv) organizational incentives and rewards v) goals and feedback vi) learning climate
	Readiness for implementation
	i) Leadership engagement ii) available resources iii) access to knowledge and information
Characteristics of individuals	Knowledge and beliefs about the intervention
	Self-efficacy
	Individual stage of change
	Individual identification with organization
	Other personal attributes
Process	Planning
	Engaging
	i) Opinion leaders ii) formally appointment internal implementation leaders iii) champions iv) external change agents executing
	Reflecting and evaluating

### Participants

This study took on purposive and snowball sampling to select participants relevant to understanding macro-level factors impacting implementation of LCIVF services in Uganda. All key stakeholders were identified based on their designation, opinion, and roles during establishment of LCIVF at MWH and were above the age of 18 years. The criteria for inclusion were any key actor or organization that was interested, engaged, or impacted by implementation of LCIVF in Uganda. A total of twenty-one study participants were included for the study, with heterogeneous representation of stakeholders, and these comprised a representative from an educational institution, clinicians (7), ART nurses (2), an international fertility specialist, hospital administrators (3), a laboratory technician, professional regulatory officials (2), a government official, a patient advocacy organization, and representatives from the construction company (2). The participants were predominantly male (*n* = 16 (76%)) and female (*n* = 5 (23%)).

### Data collection and analysis

To examine the broader contextual factors influencing implementation, we employed a case study methodology incorporating multiple data sources. Data collection methods included face-to-face and virtual semi-structured in-depth interviews, informal discussions, hospital observations, document analysis, field notes, and reflexive practices. These approaches align with the predominant data sources in case study research, specifically interviews, direct observations, and documentation, as outlined by (Yin 2003).

The first author conducted a total of 23 one-on-one, semi-structured interviews (including two participants interviewed twice) with adult participants between December 2020 and May 2021. The interviews were conducted in English, either in person or remotely via Skype, Zoom, or WhatsApp. Before the interviews, participants received a written consent document detailing the study’s purpose, procedures, anticipated outcomes, potential risks, and benefits. Participants were informed that participation was voluntary and that they could decline to participate or skip questions without penalty.

Interviews averaged 50 min in duration, were audio-recorded with participants’ consent, and transcribed verbatim for analysis. In one instance, at the participant’s request, the interview was documented through written notes by the first author. All identifying information was removed from the transcripts, and anonymized identity codes were assigned to ensure participant confidentiality.

Ethnographic observation of hospital operations at MWH was conducted from January 2021 to April 2021. A review of relevant documents, including policies, guidelines, and internal records, was performed to contextualize the implementation of ART services within the facility. Field notes were carefully recorded during hospital observations and participant interviews, capturing the author’s key observations, concepts, interpretations, and nonverbal cues to deepen insights into the determinants impacting the implementation of LCIVF services.

Data analysis was carried out using QSR International’s NVivo 12 Pro qualitative software and the CFIR as an analytical framework, iteratively to illuminate the complex, multi-level implementation process of LCIVF/affordable fertility care. Data analysis employed a hybrid approach combining inductive and deductive thematic analysis. Deductive coding was guided by CFIR, utilizing the publicly available CFIR codebook to identify factors relevant to the implementation of LCIVF in Uganda (Braun & Clarke 2006, Damschroder *et al.* 2009).

In the larger study, which explored implementation factors across micro-, meso-, and macro-levels, all CFIR domains and constructs were considered. This paper focuses specifically on macro-level implementation factors; accordingly, five CFIR constructs across two domains—outer setting (patient needs & resources, external policy & incentives, cosmopolitanism, and peer pressure) and process (engaging)*—*of the CFIR were relevant to reporting on these factors.

To allow for the emergence of novel themes, inductive thematic analysis was also conducted. Study rigor was ensured through maintaining an audit trail documenting decision-making processes and their rationales, as well as triangulating data sources (Lincoln & Guba 1985). In addition, a reflexive journal was maintained to support self-critical examination throughout the research process (Lincoln & Guba 1985). Findings were presented with direct quotations from transcripts to enhance transparency and validity in interpretation.

#### Positionality statement

As a woman of Ugandan origin, with professional experience in public health and fertility care within Sub-Saharan Africa, I approached this research with an ‘insider’ perspective, leveraging my shared identity, local knowledge, and language to build trust with participants. However, my international institutional affiliations, exposure, and positionality as a relatively young, educated woman in a traditional, pronatalist context created elements of ‘outsider’ status, which proved advantageous in eliciting open, candid discussions on sensitive topics. Balancing these dynamics, I was committed to conducting respectful, culturally attuned research, bridging the gap between academic inquiry and practical relevance while honoring the lived experiences of my participants.

## Results

This paper presents findings aligned with the relevant CFIR constructs, highlighting their influence on the implementation process. Macro-level factors identified as influential to the implementation process included *constructs* from the *outer setting* domain, namely: i) patient needs and resources, ii) external policy and incentives, iii) cosmopolitanism, iv) peer pressure, and from the process domain, namely: i) engaging. Table 3 provides a detailed table of macro-level facilitators and barriers to implementation.

**Table 3:** Detailed overview of facilitators and barriers to implementation of LCIVF initiatives in Uganda.

Domain	Constructs	Factors that facilitated implementation (enablers)	Factors that impeded implementation (barriers)
Outer setting	Patient needs and resources	Growing public demand for affordable fertility treatment Presence of patient advocacy group and demand for fertility services	Public sensitization on infertility needed to combat stigma, misconceptions and increase health seeking at fertility hospitals
	Cosmopolitanism	Partnerships with local and international organizations i.e. Makerere University, Uganda fertility society, WHO, IFFS, ESHRE, ASRM, MERCK Engagement with patient-led advocacy group i.e. JFSC Collaborated with politicians as allies to approving financing of MWH and ART bill Knowledge exchange with private sector to train medical students & embryologists	
	Peer pressure	Competitive market taken up primarily by the private sector	
	External policy and incentives	WHO declaration on infertility as a global reproductive disease UN universal declaration for human rights declaration (article 16) Development of a national ART policy International benchmarking visits to women’s hospitals and IVF departments Government funding to finance hospital construction, staff training, equipment, and information system	Regulation of financing, procurement and employment by MoF, MoH and MoPs respectively
Process	Engaging	Favourable working relationship with civil works contractor Consultation with diverse stakeholders (e.g. maternal patient organizations, lobbyists, external consultants) Engagement of media to guide journalists communicating accurate facts External consultants from India and the US provided expert advice during construction	
	i) Opinion leaders		Limited engagement with cultural and traditional leaders as social influencers
	ii) Formal appointment of internal implementation leaders	Highly engaged internal implementation team	
	iii) Champions	Former minister of health was a strong political champion Former head of obstetrics and gynecological department was an avid technical champion IVF departmental heads championed implementation efforts JFSC were a significant grassroot, patient-led organization champion	Loss of political support due to government change MoH head and priorities Discontinued engagement with patient-led organizations
	iv) External change agents executing	Consultation with diverse stakeholders (e.g. maternal patient organizations, lobbyists, external consultants) MERCK foundation provided public awareness to infertility, training opportunities and technical expertise WHO, IFFS and ESHRE also provided expert advice and technical support from LCIVF initiatives	

### Facilitators to implementation

#### Outer setting

##### Patient needs and resources

Knowledge of patient needs facilitated implementation of fertility services. Several participants cited in great detail the needs of patients during interviews, highlighting the severe impacts of infertility on one’s well-being, societal stigma due to the lack of public sensitization, absence of privacy while accessing fertility care, patient exploitation due to limited knowledge of IVF treatment, and the notable presence of patient-led advocacy groups demanding affordable fertility services at the public facility. As a clinical participant stated:

The burden of infertility is big. It is a misconception when you talk only about the high birth rate [in Uganda]. It breeds the misconception that we are fertile, and we seem to put the minority [infertile] aside. We don’t look at infertility as a disease, but infertility is one of the most painful things to deal with. Both for the practitioner but more importantly for the couple that is going through it (Clinical_ Staff 1_189–191).

Participants emphasized the major role of Joyce Fertility Support Centre (JFSC), as a civil society grassroots, patient-led organization, in bringing attention to the burden of infertility in Uganda by putting a face to it and lobbying for affordable fertility treatment.

##### External policy and incentives

***Favorable international policy & discourse on infertility:*** the evolution of global infertility discourse and policy played a critical role in the justification of implementation of affordable fertility services in the public health sector of Uganda. In particular, the UN Universal Declaration for Human Rights (Article 16) and the 2009 WHO declaration on infertility as a global reproductive disease were often quoted by participants as validation for integration of affordable fertility services. In a statement by a clinical participant, they regarded acceptance of infertility as a reproductive disease as a pivotal moment:

ART have been available for quite some time. The concept of low-cost IVF was already in plain view…The key development here [in Uganda] was first, fertility being accepted as part of reproductive health, whereas previously the argument revolved around an already high enough fertility (PA_Doctor 1_1–3).

***Government funding and oversight:*** the Government of Uganda made funding available for the construction of the MWH to the tune of USD 30.72 million through a loan from the Islamic Development Bank. Parliamentary Committee Minutes (26 July 2012) indicated that the government’s partnership with the Islamic Development Bank to construct and equip MWH would forward its efforts toward enhancing its public health system and infrastructure in Kampala. Furthermore, the Ministry of Health conducted continuous audits of the implementation process to remain abreast of progress and mitigate challenges.

***Benchmarking:*** clinical participants had opportunities to travel extensively to benchmark women’s hospitals and ART departments, as well as attend international conferences and placement visits to countries such as Ethiopia, Canada, South Africa, Egypt, and India. The female clinical pioneer behind the vision of a women’s hospital visited a similar facility in Ethiopia, which motivated her to campaign for a specialist hospital for women in Uganda.

##### Cosmopolitanism

***Partnerships and network access:*** several partnerships between MWH and external organizations played a pivotal role in establishing the hospital’s reproductive medicine department and facilitated implementation of the ART department. These network relations comprised a range of actors, both national and international, including the (ASRM), European Society of Human Reproduction and Embryology (ESHRE), International Federation of Fertility Societies (IFFS), Islamic Development Bank, Joyce Fertility Support Center (JFSC), Low-cost IVF Foundation, Makerere University, MERCK Foundation, Ministry of Finance, Ministry of Health, private fertility sector, Uganda Fertility Society (UFS), Obstetrics & Gynecological Association, Uganda Medical and Dental Practitioners Council (UMDPC), the Walking Egg, Women’s Medical Doctors Association, and the World Health Organization (WHO).

Local organizations such as Makerere University and the UFS led staff training, research, community sensitization, quality management, and lobbying for the successful securance of funding for the specialist hospital and ART department. While international entities such as WHO, IFFS, & ESHRE provided awareness, technical support, benchmark visits, conferences, and regulatory proposal development, private fertility hospitals offered practical training in ART to medical students and embryologists. The patient-led advocacy group JFSC was a pioneer and the longest-running fertility support group that rallied national and international attention to the burden of infertility and the need for affordable fertility treatment through grassroots advocacy. As one clinical participant highlighted, *‘*the organization gave a face to the disease and humanized the experiences of individuals suffering with infertility beyond statistical figures*’*.

##### Peer pressure

***Peer pressure from the private sector:*** Uganda exhibited a growing number of private fertility hospitals, generating sizable revenues because of the high treatment fees. The public health sector noticed the rise in demand and public outcry for affordable services. An administrative participant stated there was a need to counter exorbitant prices in private clinics within the public sector:

We needed to provide a service that other facilities were not offering. Many people were going to the private sector, and it was not possible for them to receive treatment because of the high charges. Furthermore, the treatment failure rates were also very high (Hospital_Admin 2_126–30).

#### Process

##### Engaging

***Political champions & allies:*** women politicians, as allies, avidly lobbied for approval of government funds to aid construction of the first women’s specialist hospital, including fertility services, with support from the fertility clinical team of MWH, the Obstetrics & Gynecological Association, and the Women’s Medical Doctors Association. The then female Minister of Health, with support from the MERCK Foundation, was an influential champion for infertility awareness and care, with statements of support from the President of Uganda. One participant described this allyship as ‘micro advocacy’, and another clinical participant recalled the critical role women parliamentarians played and the importance of giving women a voice concerning issues of their gender:

The women parliamentarians pushed back in the parliament for ultimately getting the whole idea of having a women’s hospital to be created…. They galvanized their male colleagues and others so that they get convinced when the issue is mentioned in parliament, they had a critical mass to support it…So the parliamentarians, especially women parliamentarians, were pushing at that level of government to have the women’s hospital, so we now had partners in parliament (Clinical_ Staff 3_63).

### Barriers to implementation

#### Outer setting

##### Patient needs and resources

***Poor public sensitization:*** several participants voiced that the silence and lack of public knowledge on infertility within public discourse perpetuated myths, misconceptions, and stigmatization (particularly on women), underscoring the need to combat it. In addition, the focus of public discourse on high fertility rates obscured and further marginalized persons with infertility, as noted by one clinical participant.

People need to know a lot more about infertility. They think it is witchcraft, genetic, of course in some cases it is genetic, but you know, there are all sorts of beliefs out there (Clinical_ Staff 2_295).

##### External policy and incentives

***Absence of national legislation on (in)fertility:*** the lack of national legislation on infertility and fertility care in Uganda was a notable limitation to realizing the full benefits of ART as part of the country’s national reproductive health care package. An ART bill had been drafted by the clinical team in collaboration with the professional medical association (UMDPC) in 2016 and tabled in Parliament in November 2021. The bill outlined conditions for Assisted Reproductive Technology (ART) licensure, including granting, suspending, and revoking licenses, as well as the roles of the professional body overseeing inspection and enforcement. It was inclusive, allowing treatment regardless of marital status and permitting the use and storage of donor gametes with consent, while offering flexibility regarding donor anonymity and compensation. In addition, it allowed gamete providers to offset treatment costs through gamete sharing, enabled advertising for donors and surrogates, and required contractual agreements for relinquishing rights to gametes or children born through surrogacy. However, as reported by one participant, several religious authorities and politicians protested the bill. In addition, the ART bill was combined with the Organ Donor bill, resulting in confusion and misinterpretation of IVF technologies. Together, these factors led to opposition, slowing approval of the bill.

We moved on with this bill amidst protests from some [people] Father [_], some ministers, politicians who were saying we want to distort families because we are saying if they are unable to convenience naturally, maybe you can try IVF…. So, we didn’t progress with the Bill (Participant 1_73).

Participants went on to recommend that there is a need for substantial sensitization of policy makers to facilitate consistency in understanding of ART and its prioritization.

***Diminished political support:*** midway through the implementation process, the reassignment of the then Minister of Health slowed momentum, and as participants reported, her departure as a political champion for affordable fertility care led to a decline in national-level enthusiasm and progress for the ART department.

I don’t think IVF is receiving a lot of attention even now from the ministers. I interact with them a lot, I have meetings, and I have not heard anyone talking about infertility. Dr Opendi [the then Minister] often talked about infertility, they transferred her from Ministry of Health…The new one, that one has totally different priorities (laughs) (Clinical _Staff 4_324).

#### Process

##### Engaging (opinion leaders)

***Lack of engagement with opinion leaders:*** during the implementation process, there was limited engagement with traditional, cultural, and religious leaders, as reported by participants and this frustrated implementation efforts, influencing rejection of the ART bill. These community leaders were deliberately excluded due to concern that they would block progress. As one participant described:

Of course, the religious leaders, especially the Catholic Church, say children should come normally…They see them (embryos) as lives and if thrown, is seen as abortion. We consulted them earlier, but we wanted to have the bill reach a certain stage so that we can engage them on something which is very serious. Otherwise, they can block it in the infancy because all those MPs they go to those churches (Participant_ 2_231).

## Discussion

**Figure 1:**
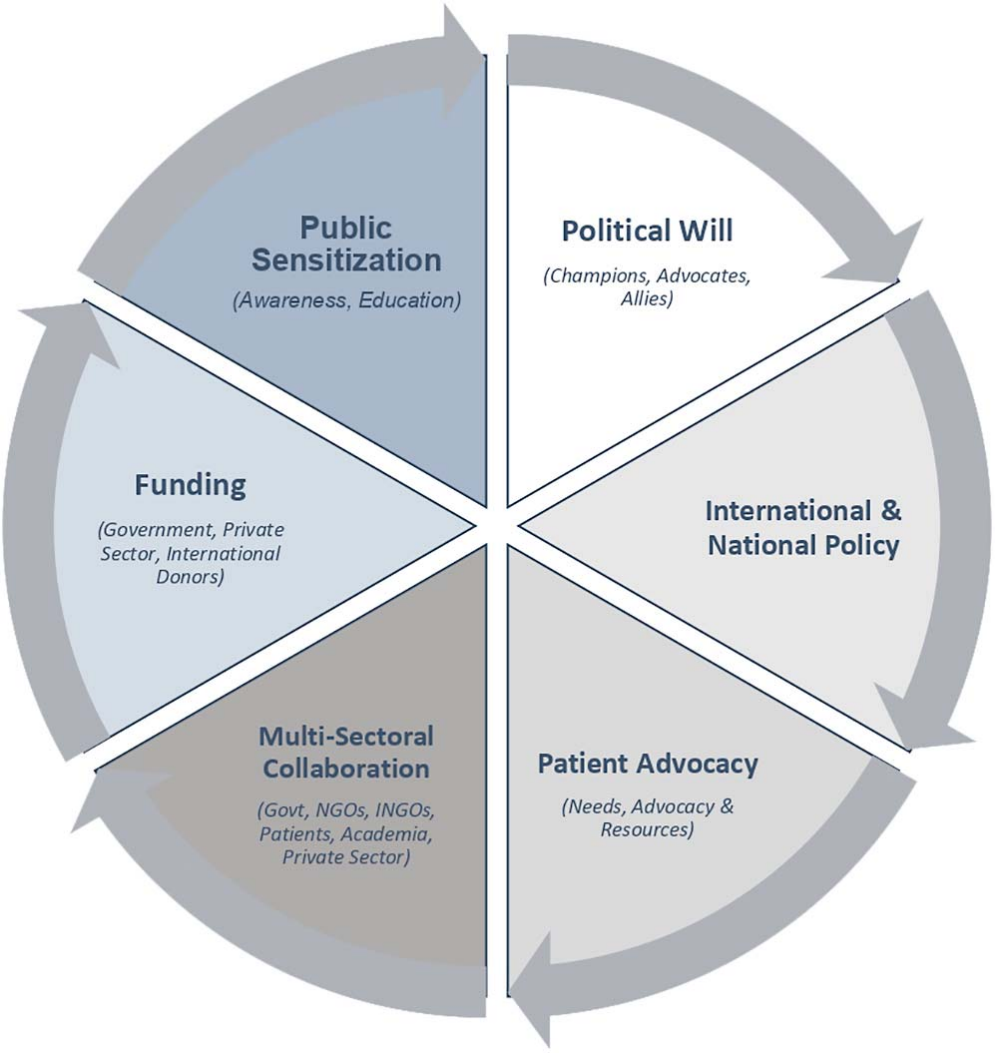
Conceptual framework for macro-level/broader implementation facilitators shaping implementation of affordable fertility care in Uganda’s public health system.

Understanding the broader, contextual-level factors that influenced the implementation of affordable fertility services in Uganda’s public health sector was one of the primary objectives of this multi-level study. An analysis of the macro-level factors that facilitated implementation, as summarized in Fig. 1 included favorable international policy on infertility, strong political support & advocacy, available government funding, multi-organizational collaboration, knowledge of patient needs & advocacy, and peer pressure from the private sector, while significant barriers consisted of lack of engagement with opinion leaders, poor public sensitization, absence of national ART legislation, and loss of a political champion.

**Figure 2:**
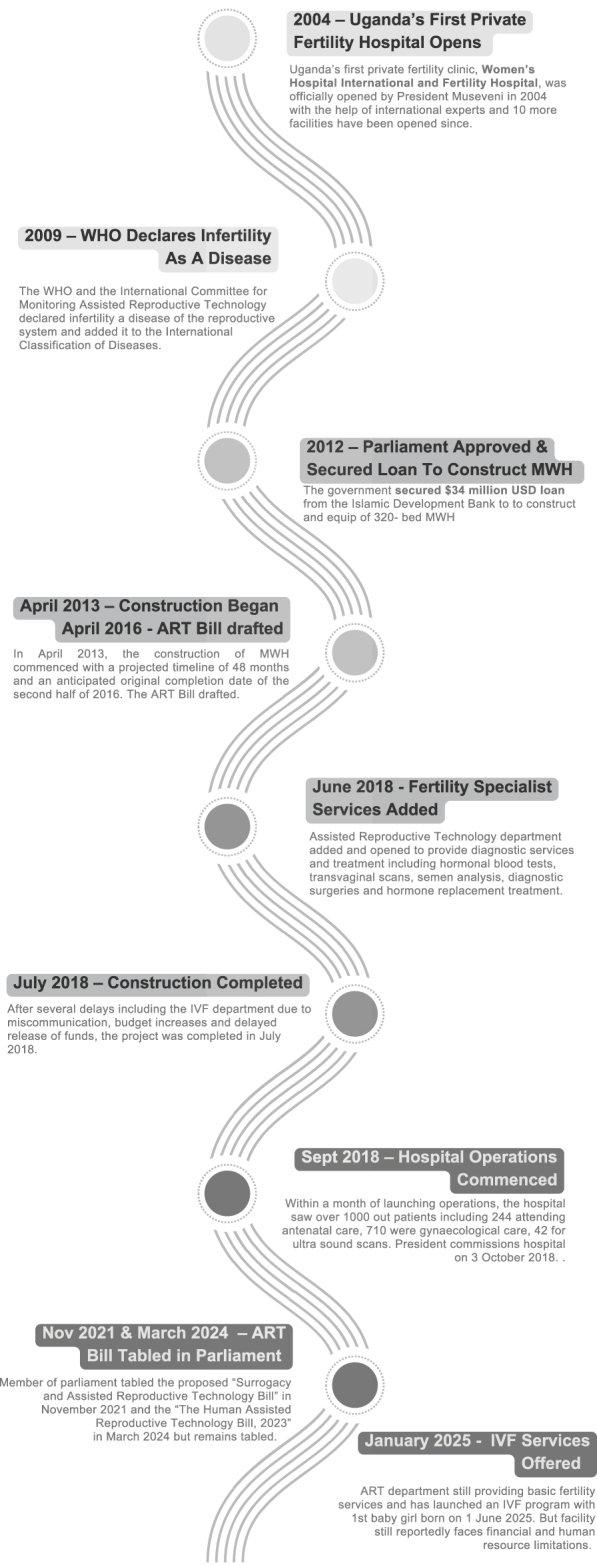
Macro-level roadmap: inclusion of affordable fertility services in Uganda’s public health system.

### External policy & incentives

The integration of fertility services into Uganda’s public health sector was majorly influenced by recognition of infertility as a reproductive disease within international reproductive health policy (external policy and incentives). Participants emphasized that the classification of infertility as a disease by the WHO and the inclusion of the right to found a family under Article 16 of the UN Universal Declaration of Human Rights (United Nations 1948, WHO 2007) provided the Ministry of Health with a crucial rationale for advancing implementation efforts. Several participants identified the ‘disease’ recognition of infertility as a pivotal development in reproductive health. These findings align with the existing literature, which underscores the role of infertility’s disease classification in facilitating its inclusion in reproductive health policies (Serour *et al.* 2019, Afferri *et al.* 2022). For instance, a study from the UK found that policymakers’ perception of infertility as a disease supported the provision of state-funded treatment through the National Health Service (NHS) (Maung 2019).

Conversely, some scholars have critiqued this framing, arguing that infertility should be viewed as a social construct shaped by societal norms around childbearing, rather than a purely medical disease (Becker & Nachtigall 1992, Greil *et al.* 2011). In this study context, defining infertility as a disease carried particular significance given the ongoing discourse on overpopulation and the nation’s high fertility rates – sentiments that may also be interpreted as socially constructed. As one participant noted, such reasoning has historically been used to justify the neglect of fertility treatment in Sub-Saharan Africa. These findings suggest that the classification of infertility as a reproductive disease by influential international organizations may serve as a critical catalyst, enabling advocacy and justification for the integration of fertility care into reproductive health services.

### Political support & funding

In this study, strong political advocacy, support, and government funding played a significant role toward implementation. The then Minister of Health, as a champion for implementation of fertility services, was a significant driver for the provision of affordable fertility services within the public health system (*political champions*). The President of Uganda, in his public addresses, expressed empathy toward the plight of the citizenry experiencing infertility. Furthermore, political allies, majority women parliamentarians, were able to galvanize support from their peers and male parliamentarians for government investment in establishing the country's first, women’s specialist hospital that included an ART department at MWH and secured over USD $30 million in loan financing to do so from the Islamic Bank & African Development Bank. This monumental milestone was reportedly a result of women’s empowerment within the Ugandan government; as one of the hospital’s pioneers stated, ‘It is important when women are given a voice to make decisions, particularly decisions pertaining to themselves because the men wouldn’t know the things that happen in women’. These results align with studies that have found similar outcomes, indicating that increased representation of women in diverse leadership roles results in positive outcomes on women’s healthcare indices (Dhatt *et al.* 2017, UN-Women 2019). Ombelet *et al.* (2008) also noted that implementation and sustenance of fertility services in low- and middle-income countries would only be possible if supported by local policymakers and the international community. Availability of government funding facilitated construction of a women’s hospital, specialist training, purchase of equipment, and installation of a hospital information system. Altogether, these observations suggest that successful implementation of fertility services requires substantial political will, empowerment of women’s voices in politics, and availability of financial resources.

### Multi-organizational collaboration

Another facilitator of implementation was the considerable engagement and collaboration by MWH with several diverse stakeholders (cosmopolitanism). Actors included both local and international organizations. For instance, the Uganda Fertility Society (UFS), along with the Uganda Medical Dental Practitioners Council (UMDPC), drafted and submitted the ART bill to support oversight of fertility practitioners in lieu of legislative approval, while Makerere University, the country’s oldest and largest public higher educational institution, offered training and research support. International organizations such as the MERCK Foundation invested in public awareness of infertility, with the then Minister of Health as the key champion, and provided technical expertise and specialist training opportunities. The ASRM, ESHRE, IFFS, and WHO provided expert guidance and knowledge transfer through professional training, benchmarking, IVF conferences/workshops, and supported the ART bill. Joyce Fertility Support Centre (JSFC), a grassroots infertility patient-led NGO, played a major role as a bottom-up driver for implementation of affordable fertility services in the public sector; organizing the first LCIVF workshop in Uganda that sparked momentum. Previous literature has highlighted the role of interorganizational networks in influencing an organization’s decision to adopt innovations (Greenhalgh *et al.* 2004). Similarly, in Ghana, transnational networks were reported to be a core contributor to implementation of IVF centers (Gerrits 2018). The study participants, however, cited that a major drawback of some organizational partnerships was that they were personal in nature (as opposed to institutional) and thus deteriorated once the linking parties moved away. For instance, reshuffling of the then Minister of Health, a champion with strong ties with the MERCK Foundation, reportedly slowed implementation progress and stakeholder engagement. Therefore, these results suggest that continuous institutional engagement with multisectoral, local, and international partnerships and engagement is beneficial to implementation and its sustainability.

### Patient needs and advocacy

In this study, patient needs and advocacy for accessible, affordable fertility services emerged as key drivers for the implementation of LCIVF services within Uganda’s public health sector (patient needs and resources). Clinical participants highlighted the long-standing demand for comprehensive fertility care, referencing their experience operating a half-day infertility clinic in a public hospital since 1991. A notable concern expressed by participants was the substantial financial burden associated with fertility treatment in the private sector, particularly its disproportionate impact on women (relative advantage).

Participants also discussed the rapid proliferation of private fertility clinics – over eight clinics established within a decade – while critiquing the exorbitant costs of treatment, reported to reach as high as USD $6,000 per IVF cycle (peer pressure). This cost was perceived as unattainable for ordinary citizens, as one participant emphasized. These findings are consistent with the existing literature that identifies the high cost of fertility care as a significant barrier to access, preventing many from benefiting from the technology to conceive (Teoh & Maheshwari 2014, Ombelet & Onofre 2019, Afferri *et al.* 2022, Dadiya 2022).

JSFC played a pivotal role in advocating for affordable fertility services both locally and internationally, notably engaging with the WHO and others. Participants underscored the organization’s visibility in humanizing the issue of infertility, which has historically been marginalized, invisible to non-affected individuals, and often reduced to statistical discourse.

These findings suggest that raising public awareness of infertility’s burden, robust patient advocacy, and clinical advocacy can serve as facilitators for implementing LCIVF initiatives within public health systems.

### Public perception, awareness and education

Participants in this study emphasized the substantial burden of infertility and its associated treatment costs, with women disproportionately affected. A major barrier to implementation and treatment uptake was limited public awareness and knowledge about infertility and its treatment modalities. Several participants called for greater public awareness to break stigma and improve treatment acceptability through public discourse. These findings are consistent with similar studies in low- and middle-income countries citing the importance of public education as part of fertility care (WHO 2010, Serour *et al.* 2019, Gerrits *et al.* 2023). For instance, in Uganda, a survey on basic knowledge of infertility of 100 women revealed it to be extremely low (Kudesia *et al.* 2018). Therefore, prioritization of public and health provider education on ART and implications of age, lifestyle, delayed childbirth, and sexual behavior on fertility is beneficial to reducing stigma and infertility and increasing utility of ART services when needed through acceptability and access to these technologies.

### Engagement of opinion leaders

Another considerable barrier to implementation was limited engagement with key community leaders, i.e., religious, cultural, and traditional leaders (opinion leaders). Community leaders, in many African contexts, are held in high regard and play a crucial role in influencing societal behavior, both at the individual and societal levels (Dennis-Antwi *et al.* 2018, Heward-Mills *et al.* 2018). These leaders could have acted as partners/champions for public sensitization, disseminating accurate information, and combatting misinformation and opposition to ART. Yet in this study, lack of engagement led to silence from community leaders and, in some cases, vocal opposition by key opinion leaders on infertility and its treatment modalities, hindering public and political support for ART. These findings contribute to the literature on the importance of engaging and educating key opinion leaders and onboarding them as champions for fertility care (Ombelet 2011, IFFS 2016). They suggest that the role of traditional, religious, and cultural leaders in similar contexts should not be dismissed or deferred, given their influence and impact on public perception toward infertility and fertility interventions.

### National ART legislation

The importance of integration of fertility care into national policy and its operationalization as part of everyday reproductive services was underscored by several participants in this study. An ART bill had been drafted by fertility clinicians and their professional governing body in adherence to the MoH’s objective to strengthen governance, quality control, and stewardship of ART. The bill addressed concerns about complex ART procedures including third-party donation, surrogacy, and clinical non-adherence penalties (e.g. loss of licensure and criminal charges). Despite these admirable efforts, limited understanding of certain aspects of ART treatment (e.g. gamete donation) by policymakers presented barriers and resistance to the ART bill’s approval. Furthermore, absence of cultural, religious, and traditional leaders’ voices as key stakeholders during this process hindered policy approval. These results are consistent with a study in Brazil on ART regulation, which found that the Catholic caucus, along with members of Congress, lobbied against highly complex ART procedures, coupling them to abortion, a sensitive topic for religious groups (Garcia & Bellamy 2015). Furthermore, a systematic review by Afferi and colleagues found that insufficient political commitment and under-recognition of the burden of infertility were barriers to integration of fertility services (Afferri *et al.* 2022). These findings indicate that inclusion of national legislation on fertility care, in combination with sensitization of policymakers and engagement with key opinion leaders can facilitate implementation and quality assurance.

While the factors above influenced the establishment of a specialist women’s hospital and an ART department, at the time of this publication, the hospital only offered basic fertility care including hormonal blood tests, transvaginal scans, semen analysis, diagnostic surgeries, and hormone replacement treatment. MWH officially launched its The IVF program in January 2025, with the first sucessful live birth - a baby girl - reported on 1 June 2025 (Wanyenya 2025, Waswa 2025). A timeline detailing the implementation process is presented in Fig. 2. Limitations to offering the full spectrum of fertility services (such as IVF and surrogacy) were due, in part, to absence of legislation to guide their implementation, additional funding for medical supplies (Nansamba 2024, Wanyenya 2025) and micro-level factors including lack of local embryologists.

### Recommendations

#### Sustained political support

This study contributes to understanding of the significance of ongoing political support and advocacy for successful implementation of affordable fertility services. More specifically, it illustrates the critical role of women politicians as allies and champions for inclusion of fertility services, garnering financial resources, and drawing up ART policy.

#### National Legislation

This study contributes to research on substantial considerations to be made in achieving national-level consensus on ART legislation amidst diverse culture and belief systems in African contexts. Early engagement of opinion leaders and stakeholders is vital to advancing societal support for fertility care, given their influence on policymakers’ decision making.

#### Multi-sectoral collaboration

Multi-sectoral, institutionally established partnerships are crucial catalysts for inclusion of fertility care in the public sector, and engagement of fertility experts, patient groups, professional associations, private sector actors, banking institutions, and international organizations offer opportunities for best practices, knowledge transfer, and financial support.

#### Public Sensitization

It is essential to prioritize public and policymaker education on infertility and treatment technologies to promote treatment acceptance and policy making that are contextually appropriate and meet the needs of the population. Patient-led organizations and community leaders (cultural, traditional, and religious) are prime actors to promote knowledge of infertility.

## Conclusion

There has been much progress toward making fertility care accessible globally. Implementation of affordable fertility services in a Sub-Saharan context, as shown in this study, is influenced by complex macro factors consisting of favorable international policy, sustained political and opinion leader support, financial resources, cross-sectoral collaboration, and public sensitization. These are important considerations for advancement of equitable access to the full spectrum of reproductive healthcare services that includes fertility care.

## Study limitations

As a qualitative single-case study relying on retrospective accounts, the potential for recall bias exists. In addition, inter-coder reliability was not conducted, as all data coding and analysis were performed solely by the author. Furthermore, the study took place during the COVID-19 pandemic, limiting opportunities for sufficient hospital observation and dynamics as attention turned towards addressing the pandemic, deprioritizing other services, including ART. Therefore, the author was unable to interview patients. Another limitation was the sensitivity of infertility as a study topic and the contentious political underpinnings of the implementation process. In fact, one of the prospective government participants asked the author why they were pursuing this research topic and declined to participate, citing it as a political minefield.

## Declaration of interest

The authors declare that there is no conflict of interest that could be perceived as prejudicing the impartiality of the work reported.

## Funding

This work was supported by the University of Waterloo, Global Health Scholarship Award and Graduate Research Scholarship Award.

## Author contribution statement

MJMN and CJ conceived the study. CJ acquired the funding for the study. MJMN performed the data collection, analysis of the data, and wrote the original draft. CJ reviewed and edited the document. All authors have read and agreed to the published version of this manuscript.
